# Osteopontin: A Key Multifaceted Regulator in Tumor Progression and Immunomodulation

**DOI:** 10.3390/biomedicines12071527

**Published:** 2024-07-09

**Authors:** Venketesh K. Panda, Barnalee Mishra, Angitha N. Nath, Ramesh Butti, Amit Singh Yadav, Diksha Malhotra, Sinjan Khanra, Samikshya Mahapatra, Priyanka Mishra, Biswajit Swain, Sambhunath Majhi, Kavita Kumari, N. N. V. Radharani, Gopal C. Kundu

**Affiliations:** 1 School of Biotechnology, KIIT Deemed to be University, Bhubaneswar 751024, India; venketeshpanda1998@gmail.com (V.K.P.); barnaleemishra12@gmail.com (B.M.); nathankita61@gmail.com (A.N.N.); diksha.ksbt@gmail.com (D.M.); sinjankhanra143@gmail.com (S.K.); samikshyamahapatra.ksbt50@gmail.com (S.M.); priyankamishra1994828@gmail.com (P.M.); swainbiswajit555@gmail.com (B.S.); smajhi034@gmail.com (S.M.); kavihumangenetics@gmail.com (K.K.); 2 Division of Hematology and Oncology, Department of Internal Medicine, Southwestern Medical Center, University of Texas, Dallas, TX 75235, USA; rameshbutti@gmail.com; 3 Biomedical Centre, Faculty of Medicine, Lund University, 223 62 Lund, Sweden; yadav.singh.amit@gmail.com (A.S.Y.); nnvradha@gmail.com (N.N.V.R.); 4 Kalinga Institute of Medical Sciences (KIMS), KIIT Deemed to be University, Bhubaneswar 751024, India

**Keywords:** cancer, osteopontin (OPN), tumor-associated macrophages, cancer-associated fibroblasts, immunomodulation, single cell transcriptomics, targeted therapy

## Abstract

The tumor microenvironment (TME) is composed of various cellular components such as tumor cells, stromal cells including fibroblasts, adipocytes, mast cells, lymphatic vascular cells and infiltrating immune cells, macrophages, dendritic cells and lymphocytes. The intricate interplay between these cells influences tumor growth, metastasis and therapy failure. Significant advancements in breast cancer therapy have resulted in a substantial decrease in mortality. However, existing cancer treatments frequently result in toxicity and nonspecific side effects. Therefore, improving targeted drug delivery and increasing the efficacy of drugs is crucial for enhancing treatment outcome and reducing the burden of toxicity. In this review, we have provided an overview of how tumor and stroma-derived osteopontin (OPN) plays a key role in regulating the oncogenic potential of various cancers including breast. Next, we dissected the signaling network by which OPN regulates tumor progression through interaction with selective integrins and CD44 receptors. This review addresses the latest advancements in the roles of splice variants of OPN in cancer progression and OPN-mediated tumor-stromal interaction, EMT, CSC enhancement, immunomodulation, metastasis, chemoresistance and metabolic reprogramming, and further suggests that OPN might be a potential therapeutic target and prognostic biomarker for the evolving landscape of cancer management.

## 1. Introduction

There were an estimated 20 million new cases of cancer and 9.7 million cancer-related deaths, according to the data from GLOBOCAN 2022 [1]. Recent data showed that female breast cancer accounts for the second most commonly occurring cancer after lung globally. The tumor core comprises of heterogenous cellular components such as stromal components consisting of cancer-associated fibroblasts (CAFs), mesenchymal stem cells (MSCs), pericytes and endothelial cells, whereas immune components consist of lymphocytes, tumor-associated macrophages (TAMs), myeloid-derived suppressor cells (MDSC) and dendritic cells (DCs), which interact with each other to form a complex tumor microenvironment (TME) [2]. These cells enhance several hallmarks of cancer via secreting a rich array of cytokines and chemokines [2].

Osteopontin (OPN), a sialic acid-rich, non-collagenous, chemokine-like, glycosylated phosphoprotein, is expressed in various cell types such as osteoblasts, osteoclasts, epithelial cells, endothelial cells and immune and stromal cells [3]. OPN has emerged as a pivotal mediator facilitating intracellular crosstalk within the breast TME [3]. Due to variations in post-translational modifications (PTMs) and proteolytic cleavage, the molecular weight of OPN ranges from 44 to 75 kDa [3]. OPN plays a crucial role in several normal physiological processes including vascularization, bone remodeling and immune regulation, as well as pathological processes including tumor progression, metastasis, immunosuppression, angiogenesis and chemoresistance [4,5]. The overexpression of OPN has been linked to poor prognosis in various malignancies, including breast, lung, glioblastoma, colorectal, hepatocellular, bladder, melanoma and acute myeloid leukemia [6]. It also serves as diagnostic as well as prognostic biomarker in different cancers [6].

**Figure 1:**
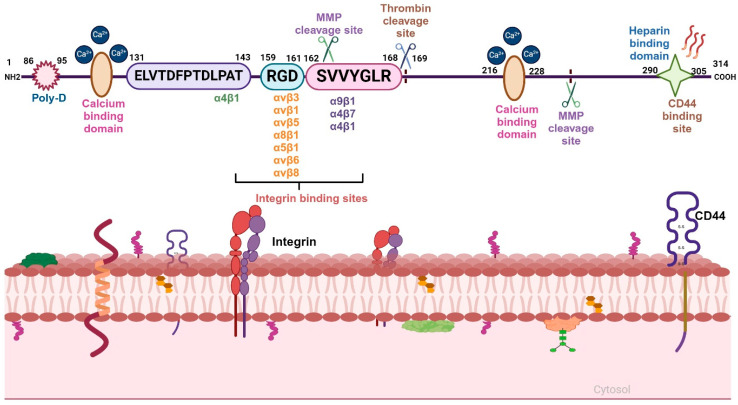
Structural domains of full-length OPN and its receptors. N-terminus of OPN consists of the poly-D region, calcium-binding domain and ELVTDFPTDLPAT sequence motif, which interacts with α4β1 integrin. The central region consists of the RGD domain, which binds with other integrins such as αvβ3, αvβ1, αvβ5, αvβ6, αvβ8, α5β1 and α8β1; the SVVYGLR sequence binds to α9β1, α4β1 and α4β7 integrins. The C-terminal region includes another calcium-binding domain, MMP-cleavage site and heparin-binding domain, which facilitate the interaction of OPN with CD44.

The diverse functional attributes of OPN in tumor progression are directly linked to its structural features as well as binding to specific integrins and CD44 (Figure 1). Moreover, OPN-mediated signaling activates various oncogenic molecules and promotes tumor growth as well as metastasis [3]. Interestingly, OPN also induces immunosuppression by activating macrophages and suppressing T-cell activation in various cancers [7]. OPN+ (SPP1+) macrophages, a subtype of TAM with a distinct feature, was recently shown to possess immunosuppressive properties and positively correlate with tumor growth and metastasis. Single cell and spatial transcriptomics analyses revealed a correlation between interactions of FAP+ CAF and OPN+ TAM with progression of colorectal cancer [8].

This review comprehensively covers recent progresses in the field of OPN with a focus on elucidating its structural features and immune modulatory properties, its role in regulating CAFs, influencing epithelial-mesenchymal transition (EMT), contributing to the enrichment of cancer stem cells (CSCs), promoting metastasis, fostering therapy resistance and influencing metabolic regulation. The goal of this review is to provide the latest progress in elucidating the role of OPN in shaping the TME. We also discuss recent treatment strategies to target OPN and its receptors for the management of cancers using small molecule inhibitors, monoclonal antibodies and aptamer-based approaches.

## 2. OPN Structure and Function

### Structural Architecture and Splice Variants of OPN

OPN is a member of the small integrin binding ligand n-linked glycoprotein (SIBLING) family of extra cellular matrix (ECM)-associated chemokine-like, aspartic acid-rich, glycosylated phospho-sialoproteins [9]. It consists of various domains, such as arginine-glycine-aspartic acid (RGD)-containing integrin binding, two calcium binding, matrix metalloproteinase (MMP) and thrombin cleavage and CD44 binding sites [10].

**Figure 2:**
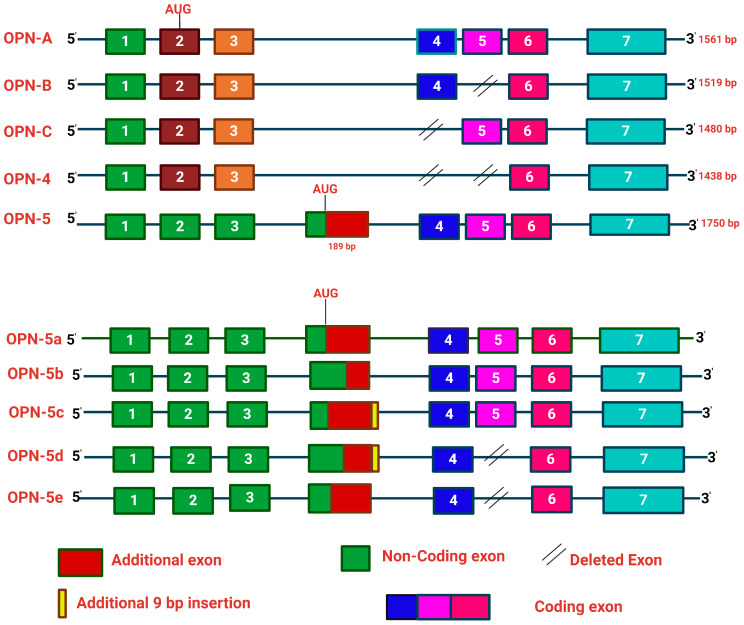
Schematic illustration of the OPN gene and its splice variants. Alternative splicing of OPN transcript results in five splice variants, which are denoted as OPN-a, OPN-b, OPN-c, OPN-4 and OPN-5. OPN-a is a full-length variant that consists of 7 exons; OPN-b lacks exon 5, while in OPN-c exon 4 is absent. In OPN-4, both exon 4 and exon 5 are missing, whereas OPN-5 is the longest variant, which consists of an additional exon, generated from a portion of intron 3. Additionally, four new sub-variants of OPN-5 (OPN-5b, OPN-5c, OPN-5d, OPN-5e) have been identified. OPN-5a is the same as OPN-5; OPN-5b has the extra shortened exon while OPN-5c has an additional 9 base pairs in the 3’ region of the extra exon. In OPN-5d, there is a deletion of exon 5 with the addition of 9 base pairs in the 3’ region of the extra exon. OPN-5e lacks exon 5.

The αvβ3, αvβ5, αvβ1 and other integrins bind OPN through the RGD motif, whereas α9β1 and α4β7 integrins interact with OPN through the SVVYGLR motif. The C-terminal region of OPN is responsible for the interaction with CD44 [11] (Figure 1). Moreover, OPN also manifests in five structural isoforms, distinguished by exon deletions, resulting from alternative splicing, exon shuffling and other PTMs [12]. PTMs including Ser/Thr phosphorylation, tyrosine sulfation and glycosylation contribute to the molecular weight variation of OPN, in the range of 44–75 kDa. These modifications induce both structural and functional alterations, deepening our understanding of the diverse roles of OPN [3]. Moreover, these five isoforms, OPN-a, OPN-b, OPN-c, OPN-4 and OPN-5, are mainly involved in various tumorigenic events [13] (Figure 2). OPN-a, the full-length isoform, consists of seven exons, whereas OPN-b and OPN-c lack exon 5 and exon 4, respectively. In addition, OPN-4 lacks both exon 4 and 5 while OPN 5 contains seven exons along with a translation start sequence positioned between the third and fourth exons. More recently, additional splice variants of OPN-5 have been reported, which are denoted as OPN-5b, OPN-5c, OPN-5d and OPN-5e [13] (Figure 2). However, OPN-5c and OPN-5d have an additional 9 bp insertion into the extra exon in between exon 3 and 4 [13]. Apart from secreted OPN (OPN-s), intracellular OPN (OPN-i) is also found to orchestrate various hallmarks of cancer. Translation of OPN-s originates at the 5’ AUG start codon while OPN-i translation begins downstream of the non-AUG codon. In addition, PTMs in OPN-i delete the 16-aa signal sequence from the N-terminus, which allows it to localize within the cytoplasm [14].

## 3. OPN Expression in Various Cancers

OPN exhibits high levels of expression in a wide range of tumor types, such as breast, ovarian, cutaneous, head and neck, thyroid, lung, liver, esophageal, gastric, pancreatic, colorectal, kidney, prostate, bladder and glioblastoma [15,16]. Tumor, stromal and tumor-infiltrating myeloid cells express high levels of OPN within the heterogeneous TME [6]. Clinical research has demonstrated a correlation between OPN expression in various tumor tissues, plasma and serum and has been shown to be correlated with an advanced tumor stage, grade, size, invasiveness, metastasis and poor survival rate of patients in a variety of human malignancies [6]. In general, OPN-a is expressed in various malignancies and is involved in promoting cancer progression, metastasis, angiogenesis, immunosuppression and drug resistance. In breast cancer, OPN-a and OPN-c are expressed at higher levels whereas high levels of OPN-a and OPN-b are expressed in lung cancer and associated with poor survival and relapse [17]. However, the correlation of OPN expression with its clinical implications in various cancers remains to be explored.

## 4. Role of OPN in Tumor Progression

OPN regulates cancer progression by influencing several hallmarks of cancers through the interaction with its receptors. This section mainly focuses on the mechanism by which tumor and stroma-derived OPN promote tumor growth.

### 4.1. OPN Receptors

#### 4.1.1. Integrin Receptors

Integrins, a heterodimeric cell surface receptor consisting of α and β subunits, are cell adhesion molecules that bind with matricellular and ECM proteins [18]. Mammals have been shown to possess 18α and 8β subunits, which constitute 24 different integrins. Both the integrin α and β subunits are type I transmembrane proteins with a short (~30–40 aa) cytoplasmic domain, substantial extracellular domain and single transmembrane domain [11,18]. OPN interacts with integrins αvβ3, α8β1, α5β1, αvβ1, αvβ6 and αvβ5 through the GRGDS motif whereas it binds with integrins α9β1, α4β1 and α4β7 via the ELVTDFPTDLPAT and/or SVVYGLR sequences [11]. The expression of integrins is low in normal adult epithelia whereas it is significantly high in most of the cancer cell types and tumor tissues [19]. The αvβ3 integrin not only binds to vitronectin but also interacts with other ECM protein such as OPN in various cell types [20]. Expression of αvβ5 is high in non-small cell lung cancer (NSCLC), prostate cancer, lung cancer, gastric cancer as well as in associated stromal cells [21]. The α5β1 integrin is an essential angiogenic marker correlating with tumor malignancy, invasiveness and development of metastasis [21]. The α8β1 integrin is mostly expressed in smooth muscle cells [22]. The α4β1 integrin is overexpressed in leukocytes including eosinophils, lymphocytes, monocytes, macrophages, NK cells, basophils and mast cells whereas the α9β1 integrin is overexpressed in macrophages and other immune cells [23]. OPN is involved in the activation of several signal transduction pathways via integrins that promote tumor metastasis, migration, adhesion and survival [24].

#### 4.1.2. CD44 Receptors

Apart from integrins, OPN also binds to CD44, a type I transmembrane glycoprotein composed of intracellular, extracellular and transmembrane domains [25]. Alternative splicing of CD44 genes results in the generation of two isoforms: CD44s (standard) and CD44v (variant) [25]. CD44v isoforms have a single variant exon as seen in CD44v6 and CD44v7, or multiple variants as observed in CD44v4-v5 and CD44v3-v10 [26]. Thrombin cleavage of OPN results in two fragments that interact with CD44 independent of the RGD sequence [27]. One of these is situated downstream of the RGD motif and has been shown to overlap with the SVVYGLR domain, because binding of OPN and CD44 competes with the α9β1 integrin but not with αvβ3 [28]. Interaction of OPN with CD44 activates multiple signaling pathways, which in turn promote tumor growth. OPN is reported to enhance the radiation resistance by maintaining stemness in the adjacent cells through activation of CD44 in glioma [29]. In addition, OPN secreted by macrophages binds to CD44 and promotes invasiveness by activating the Rac-specific guanine nucleotide exchange factor TIAM1 in bladder cancer [30]. It has been reported that OPN-CD44 interaction activates the c-Jun-NH (2)-kinase (JNK) signaling cascade, which drives the tumorigenicity in colorectal cancer [31]. Interestingly, binding of OPN with CD44 leads to cleavage of the CD44 intracellular domain by γ-secretase, which in turn governs the stemness characteristics in glioma [29]. Thus, targeting the OPN-CD44 axis might open a new dimension for cancer therapy.

#### 4.1.3. Receptor-Mediated Signaling

**Figure 3:**
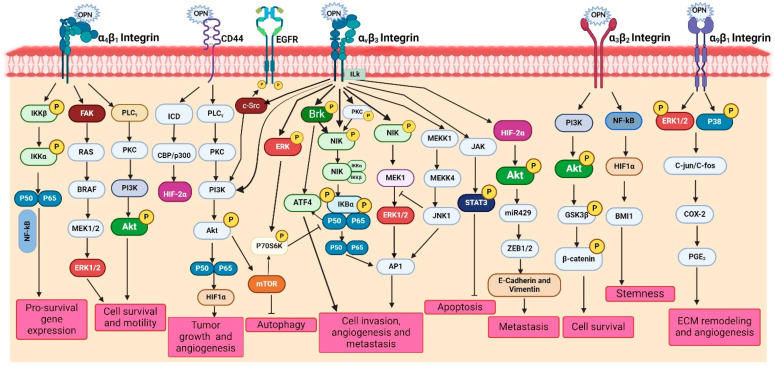
Role of OPN in the regulation of various signaling pathways. OPN through its interaction with αvβ3, α4β1, α3β2 and α9β1 integrins and the CD44 receptor transduces multiple signaling pathways and their crosstalks such as FAK/MEK/ERK, PLCγ/PKC/PI3K/Akt/mTOR, NIK/IκBα/NFκB, JAK/STAT3, PI3K/Akt/β-catenin, NFκB/HIF1α/BMI1, c-Src/EGFR/MEK/ERK and MAPK pathways. These signaling cascades induce the activation of various tumor-promoting genes such as VEGF, MMPs and COX-2, thereby inducing tumor growth at the primary sites, angiogenesis, metastases at the distance sites, ECM remodeling, immune suppression, stemness, immune evasion, chemoresistance, migration and survival.

OPN interacts with various integrins as well as other co-receptors collectively and activates downstream complex signaling cascades, such as PI3K/Akt, p38/MAPK/ERK, JNK, Ras/Raf/MEK/ERK, JAK/STAT and TIAM1/Rac1 pathways, thereby inducing the oncogenic gene expression in various cancer cells [11,15]. The activation of these pathways in cancer cells regulates several pathological processes such as cell adhesion, migration, invasion, metastasis, proliferation, tumor growth, survival, chemoresistance, stemness, angiogenesis and immune suppression. In the following section, we highlight the OPN-mediated activation of PI3K/Akt and p38/MAPK/ERK signaling pathways in various cancer cells [15]. Moreover, we depict OPN-mediated signaling pathways in Figure 3.

##### PI3K/Akt Signaling

The PI3K/Akt signaling cascade plays crucial role in regulating several cellular processes associated with tumorigenesis. Dysregulation of this pathway has been linked to tumor growth, metastasis, EMT, immunosuppression and drug resistance. OPN has been identified as a key factor that induces stem cell-like properties and cell invasion via the PI3K-Akt-GSK/β-catenin pathway in colorectal cancer cells [32]. Silencing of OPN downregulates migration and invasion but induces apoptosis and autophagy via inactivating the PI3K/Akt/mTOR pathway [33]. OPN promotes tumor progression and angiogenesis in oral cancer through the activation of the PI3K/Akt/mTOR signaling cascade [34]. Moreover, OPN-αvβ3 interaction increases HIF-1α expression, which in turn transactivates transcription factor 12 (TCF12) gene expression [35]. TCF12 engages in transcriptional repression of the VE-cadherin by interacting with histone deacetylases and enhancing zeste homolog 2 (EZH2), promoting the endothelial-mesenchymal (EndoMT) transition [35]. OPN expression is regulated by the EGF/PI3K signaling pathway in HepG2 cells [36]. Additionally, OPN upregulates angiogenesis via activation of PI3K/Akt and ERK1/2 pathways in breast cancer, whereas it promotes tumor progression via PI3K/Akt/Twist signaling axis in HCC [37]. OPN knockdown reduces Akt phosphorylation and downregulates the expression of VEGF and MMP-2 in gastric cancer [38]. Silencing calpain subunit 4 (Capn4) downregulates OPN expression and suppresses the migration of ovarian cancer cells [39]. Interestingly, OPN has also been reported to modulate drug resistance through the PI3K/Akt pathway in several cancers. For example, OPN overexpression increased PI3K, p-ERK1/2, and excision repair cross-complementation group 1 (ERCC1) expressions in lung cancer and caused cisplatin resistance, but OPN silencing decreased this effect [40]. Similarly, blocking the OPN-mediated PI3K/Akt signaling pathway reversed OPN-induced cisplatin resistance in HCC cells [41]. Similarly, OPN is upregulated in EGFR-TKI-resistant NSCLC cells, thereby activating the PI3K/Akt pathway, leading to downregulation of EGFR-TKI-induced apoptosis while augmenting EMT [42]. Moreover, epoxyazadiradione, a limonoid, inhibits the growth of breast tumors by depolarizing the mitochondria and inducing caspase-dependent apoptosis through the inhibition of the PI3K/Akt pathway [43]. A splice variant of OPN, OPN-c, has been reported to be involved in promoting tumorigenesis and proliferation by activating the PI3K/Akt pathway in ovarian cancer [44] (Figure 3).

##### p38/MAPK Signaling

The binding of OPN to its receptors activates the MAPK pathway, which in turn regulates several vital processes including EMT, chemoresistance and senescence [15]. It is observed that OPN-induced PI3K/Akt and MAPK/Erk1/2 cascades promote EMT, which in turn enhances cancer cell proliferation and migration in lung cancer cells [45]. OPN-mediated stimulation of the MAPK pathway is essential for the cell growth and metastasis in HCC [46]. In prostate cancer, binding of OPN to αvβ3 stimulates VEGF expression via the MAPK pathway, resulting in enhanced cancer cell proliferation and invasion [47]. However, in gastric cancer, OPN stimulates NF-κB nuclear translocation via the MAPK and PI3K/Akt pathways, which in turn increases HIF-1α to support the proliferation and survival of cancer cells [48]. It was revealed that after deletion of OPN in breast cancer cells they exhibited increased levels of apoptosis induced by cyclophosphamide compared to controls [49]. Immediate early response 2 (IER2) triggers senescence in cancer cells via the p53/MAPK/Akt-pathway and results in poor prognosis in melanoma patients [50]. Doxorubicin-treated breast cancer cells inhibit caspase-3-induced apoptosis via activating the MAPK pathways in response to OPN [51] (Figure 3).

##### Other Signaling

Many other signaling pathways are also involved in OPN-mediated regulation of tumor progression, angiogenesis and metastasis. For instance, OPN interacts with CD44 and integrins and mediates several signaling networks, such as the JAK/STAT and NIK pathways, to trigger gene expression that mediates invasion, metastasis and angiogenesis. Intracellular signaling pathways can be triggered by the c-Src-dependent transactivation of EGFR through interaction with OPN and integrin via the Brk/NF-κB signaling pathway, which ultimately regulates VEGF expression in breast cancer cells [52]. Under hypoxic conditions, OPN triggers integrin-linked kinase (ILK)/Akt-mediated NF-κB activation, which results in HIF-1α-dependent VEGF expression in breast cancer cells and subsequent angiogenesis [15]. NF-κB and HIF-1α are downstream of OPN signaling and induce a CSC-like phenotype in HCC [6]. OPN via αvβ3 integrin induces JAK2/STAT3 activation in MDA-MB-468 and MCF-7 cells, resulting in breast tumor growth and angiogenesis [53]. OPN expression through Akt/mTOR and MNK/eIF4E pathways triggers infiltration of suppressive MDSCs, thereby creating an immune-suppressive TME and promoting tumor proliferation in prostate cancer [54]. OPN, by activating the JAK1/STAT1 pathway, promotes bladder cancer progression and metastasis [55]. OPN signaling can activate anti-apoptotic and pro-survival pathways through PI3K-Akt and NF-κB signaling and enhances angiogenesis, migration and metastasis in gastric and liver cancers [56]. In addition, OPN regulates MAPK and PI3K-dependent NF-κB activation, leading to gastric cancer progression [57]. Mechanistically, OPN induces ROS production by upregulating NADPH oxidase 1 (NOX1) expression, whereas knockdown of NOX1 partially reduces the OPN-induced cell proliferation and migration. Moreover, blocking the JAK2/STAT3 activation significantly decreases the OPN-induced NOX1 transcription [58]. Furthermore, OPN-CD44 interaction enhances chemoresistance and induces the ABC drug efflux transporter through activation of the PI3K/AKT signaling pathway in ovarian cancer cells [59]. IL-6 derived from CAFs promotes progression of head and neck cancer through the OPN-αvβ3-NF-κB axis [60]. The abnormally activated OPN/integrin αvβ3/FAK signaling axis is responsible for EGFR-TKI resistance in EGFR mutant NSCLC [61]. Similarly, OPN derived from TAMs upregulates PD-L1 expression and predicts poor prognosis in NSCLC [62] (Table 1, Figure 3).

### 4.2. Multifaceted Functions of OPN in Tumor Progression

**Figure 4:**
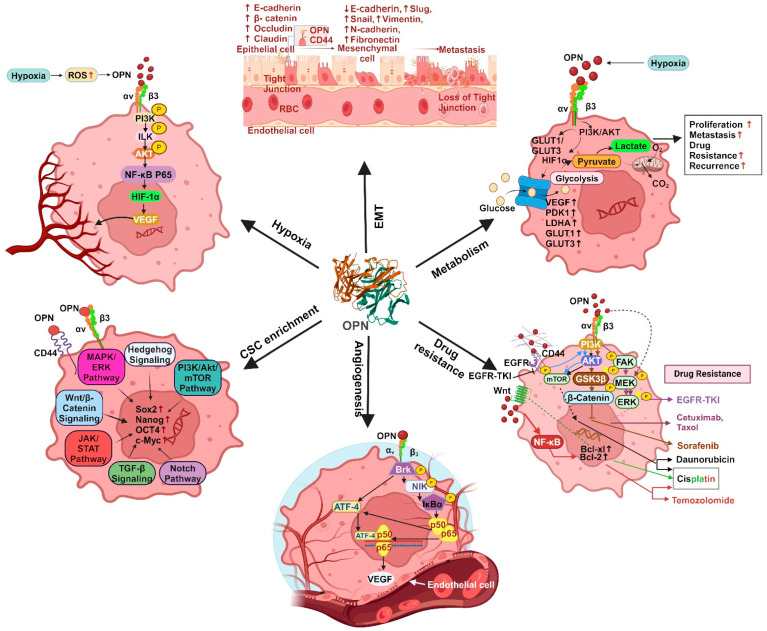
Diagrammatic representation of multifaceted function of OPN in various tumors. OPN regulates EMT, resulting in the loss of tight junctions, thereby enhancing metastasis with the low expression of E-cadherin and high expressions of N-cadherin, vimentin, slug, snail and fibronectin. Under hypoxic conditions, OPN induces activation of PI3K leading to phosphorylation of Akt, thereby upregulating VEGF-dependent angiogenesis. OPN is responsible for metabolic function by activating HIF1α under hypoxic conditions, which further aids in the glycolytic process with high expression of VEGF, PDK1, LDHA, iNOS, GLUT1 and GLUT3. OPN further regulates CSC enrichment by activating a cascade of signaling pathways involving PI3K/Akt/mTOR, hedgehog, MAPK, Wnt/β-catenin, JAK/STAT and notch signaling. The interaction between OPN, αvβ3 and CD44 results in the activation of PI3K/Akt, FAK/MEK/ERK, EGFR and Wnt/NFκB signaling cascades, thereby aiding therapeutic resistance.

OPN has been shown to stimulate the progression of cancer by activating specific signaling pathways. These components collectively create an intracellular signaling traffic network that controls the expression of various oncogenic molecules that are essential for initiating tumorigenesis, regulating EMT, stemness, angiogenesis, metastasis and drug resistance. The OPN-regulated various oncogenic functions are depicted in Figure 4.

#### 4.2.1. EMT

EMT is a multifaceted process that occurs when epithelial cells lose cell-cell and cell-matrix adhesion capacity and transform into a mesenchymal phenotype. The decrease in epithelial integrity is characterized by upregulation of fibronectin, vimentin and N-cadherin and downregulation of E-cadherin [11]. OPN modulates many transcription factors that regulate EMT such as Slug, Twist and Snail, in various solid cancers including breast. OPN also plays a crucial role in initiating EMT via triggering twist activation in breast cancer [67]. Interestingly, Butti et al. have demonstrated that OPN-educated fibroblasts induce EMT in MDA-MB-231 and MCF-7 cells through the secretion of C-X-C motif chemokine ligand 12 (CXCL12) and promote breast cancer progression [63]. Moreover, OPN triggers an autocrine MAPK intracellular signaling cascade that leads to activation of Twist and upregulation of Bmi1 in MDA-MB-231 cells [68]. Similarly, Snail is also involved in the upregulation of genes linked with mesenchymal and invasive characteristics [67]. OPN interacts directly with runt-related transcription factor 2 (Runx2) resulting in induction of Snail-dependent EMT in mammary epithelial cells [69]. The OPN-specific aptamer reduces Snail expression, thereby abrogating EMT using in vitro breast cancer models [67]. In addition, OPN induces NF-κB activation and increases the expression of both zinc-finger E-homeobox binding transcription factors (ZEB1) and ZEB2, resulting in the acquisition of an EMT-like phenotype in breast cancer cells [70]. The intricate interplay between the tumor and its surrounding environment is a crucial regulatory component of EMT and OPN is proven to be a key player in the tumor-stroma interaction. Tumor-derived OPN upregulates COX-2 expression in TAMs, leading to an enhancement in angiogenesis and melanoma growth [64]. Myofibroblasts produced directly by OPN or by other mechanisms secrete a variety of chemokines, TGFβ, IL-6, sphingosine-1-phosphate (S1P) and angiotensin II (Ang II), which promote EMT [71]. In both in vitro and in vivo breast cancer models, tumor-derived OPN has been shown to regulate the transformation of tissue-resident normal mammary fibroblasts into tumor-supporting CAFs [72].

Both tumor-derived and exogenous OPN can trigger the transition of MSCs to CAFs in breast cancer models. This is accomplished by activating MSCs to generate TGFβ, thereby initiating a feedback loop that drives the CAF phenotype [73]. OPN is found to upregulate HIF-1α, which leads to twist 1 activation followed by EMT. Remarkably, OPN-s initiates cancer metastasis by inducing the EMT, whereas OPN-i activates the mesenchymal-to-epithelial transition (MET) to promote metastatic formation [74]. Therefore, targeting OPN/αvβ3 integrin and the OPN/CD44 signaling cascade may control epithelial-mesenchymal plasticity in various cancers (Figure 4).

#### 4.2.2. Enrichment of CSCs

CSCs, also known as tumor-initiating cells, are a subpopulation of cells within a neoplasm that exhibit distinctive characteristics, including self-renewal capacity, pluripotency and the ability to generate distinct progeny, mirroring the heterogeneity inherent in the primary tumor [75]. These cells possess a unique ability to sustain tumorigenesis by maintaining their undifferentiated state, resisting conventional anti-cancer therapies and orchestrating the hierarchical organization of the tumor mass [75,76,77]. CSCs are majorly characterized by the expression of CD44, CD24, ALDH1 and CD133 in breast cancer [75].

Several factors secreted by tumors and stroma, cell-cell contacts or cell-matrix interactions are reported to impact the stemness of cancer cells through autocrine, paracrine and juxtracrine mechanisms [11]. OPN emerges as a critical player in the enrichment of CSCs due to its ability to interact with its receptor and a recognized stem cell marker, CD44 [78]. OPN, along with the stem cell marker CD44, shapes a perivascular niche that promotes the CSC phenotype and radiation resistance in glioma. The γ-secretase-cleaved intracellular domain of CD44 interacts with OPN that enriches stem cell phenotype and glioma growth through CBP/p300-dependent activation of HIF-2α [29]. Furthermore, ALDH^hi^CD44^+^CD24^−^ stem cells successfully metastasize to bone, where bone-derived OPN is implicated in promoting the stem-like phenotype in breast cancer cells, thereby influencing metastasis. The mechanism underlying OPN-induced CSC-facilitated metastasis involves CD44 and RGD-dependent cell surface integrins, which augment the functional response to bone-derived OPN, potentially through activation of WNK-1 and PRAS40-related pathways [79]. Thus, bone metastasis can be targeted by disrupting this dynamic interaction. However, another study reported the contrasting role of osteoclast-derived OPN in bone metastasis, suggesting a counterintuitive option for the treatment of breast cancer-associated bone metastasis [80]. Mechanistically, the low-density lipoprotein receptor-related protein 5 (Lrp5)-overexpressing osteoclast-derived OPN regulates Hsp90ab1 (Hsp90 beta) and moesin (MSN). Importantly, Hsp90ab1 immuno-precipitates latent TGFβ and inactivates TGFβ, whereas MSN interacts with CD44, thereby inhibiting the CD44 pathway [80]. This interaction suggests a potential impact on CSCs, highlighting the complexity of the regulatory network established by OPN. The dichotomous role of OPN signaling may arise from the existence of multiple isoforms of both OPN and its receptors, each exhibiting context-dependent functions. Hu et al. demonstrated that CD44v exhibits significantly higher lung metastatic potential as compared to CD44s in the CD24^−^/CD44^+^ CSC population [81]. Modulating the CD44v/CD44s ratio through epithelial splicing regulatory protein 1 (ESRP1) expression influences lung metastasis without affecting the stemness. CD44v, responsive to OPN in the lung environment, enhances cancer cell invasiveness and promotes lung metastasis, distinguishing it from CD44s [81]. These findings identify a subset of metastatic breast CSCs marked by CD44v expression and responding to OPN, suggesting CD44v and OPN to be key regulators of CSCs and metastasis (Figure 4).

#### 4.2.3. Chemoresistance

Chemoresistance can be induced by the interplay between the heterogenous cell population within the TME and orchestrated by tumor-initiating cells (TICs) or CSCs [82]. This interplay between several intrinsic factors such as tumor mutation load, heterogeneity, ECM and epigenetic modifications, along with extrinsic factors such as pH, hypoxia, paracrine signaling and other stromal cells, triggers chemoresistance [83]. Mechanistically, chemoresistance results from upregulation of various multidrug resistance efflux pumps such as ABCC 1-9, ATP binding cassette (ABC) transporters and P-glycoprotein (P-gp) [84]. An elevated level of OPN mRNA expression was associated with poor disease-free survival (DFS) and overall survival (OS) in a large cohort of breast cancer patients treated with adjuvant chemotherapy in clinical trials [85]. Overexpression of ABC transporters is reported to be a key factor in drug resistance as it can mediate the efflux of various drugs, thereby decreasing the intracellular concentration of the drug. Stromal OPN increases ABCG2 expression and enriches the side population (SP) through the ERK2-dependent pathway in melanoma [86]. In prostate cancer, it has been shown that the activation of FAK leads to increased expression of P-gp upon the binding of secreted OPN to αvβ3 integrin [87]. Furthermore, Yi et al. have shown that binding of OPN to αvβ3 activates the PI3K/Akt/GSK3β/β-catenin signaling cascade, resulting in cell survival and sorafenib insensitivity in FLT3-ITD mutant AML cells [88]. OPN causes aberrant activation of the PI3K/Akt signaling pathway via CD44 and αvβ3 integrin, while blocking OPN could reverse the chemoresistance of cisplatin in HCC [41]. Upon binding to αvβ3, OPN induces autophagy, thereby maintaining FOXO3a stability, which increases tumor growth and resistance to epirubicin and cisplatin in HCC cells [89]. Hyperactivated EMT has also been shown to be linked with increased treatment resistance in cancer cells and this may be induced by abnormal activation of multiple signaling pathways that drive the EMT phenotype [90]. Moreover, OPN induces the expression of N-cadherin, vimentin, Twist, Slug and MMP9 by activating the GLI-dependent hedgehog signaling pathway in breast cancer. In this study, the authors reported that OPN upregulates the expression of drug-resistant related proteins such as ABCB1 and ABCG2, which in turn promotes the EMT and induces the efflux of therapeutic drugs such as paclitaxel, cisplatin and doxorubicin [91] (Figure 4).

#### 4.2.4. Angiogenesis

Angiogenesis, a pivotal process in tumor growth, is intricately regulated by VEGF. It has been shown that OPN secreted by the tumor cells can enhance their metastatic potential and angiogenesis by regulating VEGF [92]. For example, upregulation of OPN by TBX3iso1 in breast cancer cells leads to angiogenesis using in vivo mice models [93]. Disintegrin and metalloproteinase 8 (ADAM8) activates OPN expression through the JAK/STAT3 pathway, thereby enhancing angiogenesis in U87 cells and primary macrophages [94]. In addition, high levels of ADAM8, a proteolytically active member of the ADAM family found in several malignancies, contribute to tumor cell migration and invasion and are associated with poor patient prognosis [94]. Chakraborty et al. have shown that OPN promotes VEGF-dependent angiogenesis via the activation of the Brk/NF-κB/ATF-4 signaling pathway in paracrine, autocrine and juxtracrine manners in breast cancer [65]. Moreover, under hypoxic conditions, OPN modulates HIF1α-induced VEGF expression via the ILK/NF-κB signaling cascade, which ultimately culminates in breast cancer progression and angiogenesis [95]. OPN triggers the inducible T cell costimulator ligand (ICOSL) and thereby promotes angiogenesis and cell migration in breast cancer [96]. The interaction between OPN+ macrophages and endothelial cells promotes angiogenesis through VEGF-A-VEGFR1/R2 [97] (Figure 4).

#### 4.2.5. Metastasis

In several cancers, there is a correlation between high levels of OPN expression and metastasis. OPN facilitates the molecular process that dictates the development of metastatic lesions, including inhibition of apoptosis, ECM degradation and remodeling, cellular migration, host immune cell evasion and neovascularization [98]. Breast cancer cells spontaneously metastasize to lung in mouse models and the level of OPN is high in lung lesions [99]. In small-cell lung cancer, lymph node metastasis is positively correlated with serum OPN level, suggesting its role as predictor of OS [100]. Tumor-associated cell-derived OPN enhances the CD44v6 expression in colon CSC through the Wnt/β-catenin pathway, thereby promoting metastasis [101]. In gastric cancer, an increased OPN level is correlated to lymph node and distant metastasis [102]. The SNP of an OPN promoter at locus -443 and associated haplotypes (Ht2 and Ht3) considerably boost the promoter activity and OPN expression, which in turn increases tumor proliferation and lung metastasis in HCC [103]. In osteosarcoma, lysosomal-associated membrane protein 3 (LAMP3) may control the invasion and metastasis by modulating downstream signaling of OPN [104]. Loss of OPN governs bone metabolism via modulating the miR-34c/Notch1 pathway, which helps to prevent osteolytic bone metastases in NSCLC [105]. The osteolytic bone metastasis in breast cancer is significantly inhibited in conditional knockdown of OPN in nude rat models [106]. The neutralization of OPN effectively mitigates the enhanced osteoclast development and bone metastasis induced by Fam20C deficiency [107]. Combination of erufosine with OPN knockdown enhances anti-metastatic effects for the control of skeletal metastases using breast cancer cells [106]. Breast cancer bone metastasis is thought to be significantly influenced by Runx2, which is regulated by OPN-αvβ3/CD44 axis [108]. The premetastatic niche in bone marrow requires OPN-dependent migration of CAF to promote stemness using an in vivo breast cancer model [109] (Figure 4). Therefore, OPN is a vital regulator of bone and lung metastases in breast cancer.

#### 4.2.6. Cancer Cell Metabolism

Through metabolic alterations linked to carcinogenesis, transformed cells can persist in abnormal growth and invade various tissues by avoiding tissue homeostasis and utilizing an array of internal signaling pathways along with a variety of local tissue and whole-body resources. Significantly, the stromal cells in the TME, and the transformed cells themselves, all undergo metabolic remodeling in various cancers [110]. This promotes the accumulation and dissemination of cancer cells, weakens the immune system to prevent tumor growth, and increases the lethality associated with cancer [110]. This can be accomplished by rewiring the glucose metabolic pathways, which confers drug resistance and facilitate metastasis [111]. Tumor cells require glucose as a metabolic energy source for survival and proliferation. Glucose transporters (GLUTs) facilitate aerobic glycolysis, commonly called the Warburg effect, by delivering glucose into the cytosol. The class I glucose transporters GLUT1 and GLUT3 are sensitive to hypoxia and have a strong affinity for glucose [112]. Numerous proteins including OPN, VEGF, pyruvate dehydrogenase kinase 1 (PDK1), iNOS, lactate dehydrogenase A (LDHA), EPO, GLUT1 and GLUT3 are regulated by HIF-1 under hypoxic conditions [113,114]. It has been reported that GLUT1 and GLUT3 upregulation is linked to a poor prognosis in breast cancer [115]. Additionally, poor survival in NSCLC is associated with overexpression of GLUT1 [116]. OPN is upregulated in hypoxic environments, which leads to increased expression of GLUT1 and GLUT3 via αvβ3 integrin-mediated PI3K/Akt and p38/MAPK pathways. This process ultimately results in the development of osteosarcoma [112] (Figure 4).

## 5. OPN-Mediated TME Regulation

**Figure 5:**
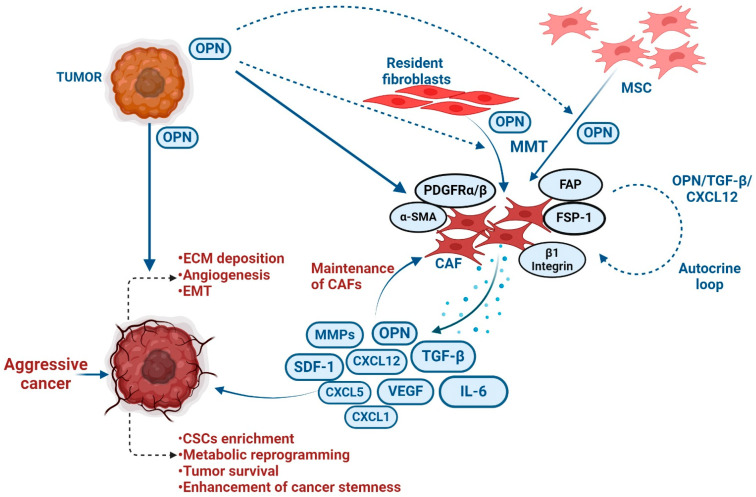
Differentiation of resident fibroblasts and MSCs into myofibroblasts by tumor-derived OPN. Tumor-derived OPN is involved in the transition of resident fibroblasts and MSCs into myofibroblast or CAFs. CAF-derived factors induce ECM deposition, EMT, angiogenesis, CSC enrichment, metabolic reprogramming and tumor survival, resulting in the enhancement of tumor progression.

The ECM, stromal cells, immune cells, matricellular proteins, fibroblasts, cytokines and growth factors define the complex microenvironment surrounding tumors. Recent evidence suggests that tumor cell-TME interaction modulates tumorigenesis, tumor cell invasion, metastasis, chemoresistance and immune response (Figure 5).

### 5.1. CAF

The reciprocal interaction between tumor cells and fibroblasts plays a crucial role in the progression of breast cancer [117,118]. OPN emerges as a key regulator in facilitating the crosstalk between tumor and stromal fibroblasts, contributing to breast cancer advancement [76,117,119]. Sharon et al. demonstrated that OPN has the ability to reprogram normal fibroblasts, inducing a proinflammatory state that supports the growth of breast cancer [120]. In this study, the authors revealed that the OPN-reprogrammed fibroblasts secrete various proinflammatory cytokines, including CXCL1, CXCL2, COX-2 and IL-6. The ability of OPN to reprogram mammary fibroblasts was found to be reliant on signaling through CD44 and αvβ3 integrin [120]. Butti et al. have reported that tumor cell-derived OPN triggers the differentiation of fibroblasts into CAFs through activation of Twist1 [63]. Moreover, OPN induces Twist1-dependent myofibroblastic protein expression, such as α-smooth muscle actin (α-SMA), fibroblast-specific protein (FSP), fibroblast activation protein (FAP), stromal cell-derived factor-1 (SDF1) and platelet-derived growth factor β (PDGFR-β) by binding to CD44 and αvβ3 integrins, activating Akt and ERK signaling pathways. OPN-driven CAFs then release CXCL12, inducing EMT and angiogenesis. OPN and CXCL12 are identified as crucial components perpetuating this crosstalk [63]. Costa et al. have identified FAP+ CAFs (CAF-S1) subsets enriched mainly in triple-negative breast cancer (TNBC), which induces the immunosuppressive microenvironment by secreting CXCL12 [121]. The induction of FAP and CXCL12 in CAFs by OPN suggested that the re-programming of CAFs may play an immunosuppressive role through CXCL12 secretion. Furthermore, studies also implicated the involvement of OPN autocrine signaling in the generation of CAFs using the loss-of-function studies in fibroblasts [63,120]. To support this observation, it has been indicated that CAFs actively secrete OPN, while its silencing in α-SMA+ CAF attenuates the growth of colonies using breast cancer cells [122]. The 3D cell culture and animal model data revealed that blocking CAF-derived OPN effectively prevented lung metastasis in breast cancer. Intriguingly, this study also highlighted a correlation between OPN expression and tumor invasiveness in patient specimens [123]. Collectively, the studies demonstrate that OPN plays an important role in CAF and cancer cell interaction leading to breast cancer progression.

The senescent fibroblasts exhibit characteristics reminiscent of myofibroblasts (CAFs), serving as potential regulators of senescence-associated cancers. Senescent fibroblasts induce pre-neoplastic growth through OPN [124]. Interestingly, the reduction of OPN levels through RNAi does not affect the induction of senescence in fibroblasts, however, it has a profound impact on diminishing the growth-promoting activities of senescent fibroblasts [125]. Another study has shown that altering Tiam1 expression in senescent fibroblasts induces the invasive and migratory potential, EMT, and CSC characteristics by upregulating OPN in breast cancer cells [123].

Although resident fibroblasts contribute majorly to CAF population, generation of CAFs from different cellular sources including MSCs has been well documented [126]. Tumor-derived OPN has also exhibited significant role in the generation of CAFs from MSCs in breast cancer. OPN induces expression of various myofibroblast markers including α-SMA, FSP-1 and CXCL12. OPN-educated MSCs also produce higher levels of CXCL5 through integrins and activate c-Jun, and OPN-interacted MSCs induce breast cancer metastasis [127] (Figure 5). Moreover, OPN instigates integrin-dependent MSC expression via TGFβ1 to facilitate acquisition of the CAF phenotype. OPN activates the TGF-β expression transcriptionally via the myeloid zinc finger 1 (MZF-1) transcription factor. This study concluded that tumor-derived OPN induces MSC-CAF trans-differentiation to enhance tumor growth and metastasis via the OPN–MZF1–TGF-β1 signaling pathway [73]. These studies show that OPN may be a potential fibrogenic factor in the breast tumor microenvironment that facilitates the growth and metastasis of cancer cells.

### 5.2. Adipocytes

Adipose tissue (AT) is the site of inflammatory responses linked to obesity, which trigger a range of cytokines and modify metabolic regulation [128]. It has been demonstrated that OPN causes inflammatory signaling in adipocytes and is primarily produced by macrophages in obese AT [129]. Obese humans and mice exhibit elevated levels of OPN in plasma circulating levels as well as in AT macrophages [130]. MMP-2 and MMP-9 are highly expressed in various cancer cells and targeting MMPs with their inhibitors may act as an important therapeutics in cancers [131].

### 5.3. Osteoclast

Osteoclastic cells originating from monocytic lineage are involved in bone remodeling, bone resorption and ossification [132]. About 15-30% of breast cancer patients are prone to their cancer metastasizing to bone [107]. Zuo et al. have shown that neutralization of OPN downregulates the Fam20C deficiency, which reduces differentiation of osteoclast along with bone metastasis [107]. This study has established a correlation between OPN and osteoclastogenesis and suggests that OPN may act as a potential therapeutic target for breast cancer bone metastasis.

## 6. OPN in Immunomodulation

Within the heterogenous TME, the tumor immune microenvironment (TIME) comprises spatially distributed immune cells such as lymphocytes, monocytes, dendritic cells and macrophages. These cells play a critical role in supporting tumor progression and metastasis via crosstalk with the cancer cells. The role of OPN in immunomodulation has been greatly appreciated in mediating tumor-immune cell interaction and creating an immunosuppressive TIME. OPN can reprogram immune cells such as macrophages to induce tumor growth, angiogenesis and metastasis by secreting pro-tumorigenic cytokines and growth factors. Further, these re-educated immune cells may also secrete OPN, which further aids in tumor progression [11]. Besides this, OPN can also induce an immunosuppressive effect in TME by promoting anti-inflammatory macrophages and inhibiting T cell activation via various mechanisms [11].

### 6.1. OPN Modulates Macrophages into TAMs

Various experimental studies have identified a population of macrophages in TME known as TAMs linked with drug resistance and poor prognosis in several cancers. TAMs are categorized as proinflammatory M1 and anti-inflammatory M2 phenotypes. M1 macrophages are known to induce inflammatory cytokines for the anti-tumor Th1 cell response, whereas M2 macrophages are involved in promoting tumor growth, angiogenesis, metastasis, CSC regulation and immune suppression in cancer by secreting anti-inflammatory cytokines [133].

The unique structure and ability of OPN to bind integrins make it an efficient signaling molecule and it predominantly promotes tumor progression by governing macrophage polarization, activation, migration and immunosuppression [134]. Evidence suggests that OPN+ macrophages are a critical determinant of tumor progression within the TME, but the comprehensive regulatory network underpinning tumor regulation remain unclear. OPN induces polarization of macrophages into M2 type, characterized by CD163, CD206 and CD209 expression, to promote tumor progression. It also induces immunosuppressive ques in TME mediated through the TAMs [6]. Treating monocytes with OPN-rich conditioned media from cancer cells leads to an increase in M2 macrophages, and a co-implanted xenograft of OPN-expressing tumor cells and monocytes leads to enhancement of tumor growth and poor survival in gastric cancer mouse models [135]. However, this effect of OPN in promoting M2 macrophages may not be universal since treatment of monocytes from healthy donors with recombinant OPN does not enhance the M2 population although it helps in the maintenance of the M2 phenotype [136]. Besides promoting M2-type macrophages, OPN also plays a crucial role in recruitment of TAMs in TME by acting as a chemoattractant and inducing TAM migration. A study using OPN KO mice demonstrated that absence of OPN reduces the infiltration of macrophages in tumors while there is no effect in normal tissue [64]. Another report suggests that reduced macrophage infiltration and enhanced T cell activity are observed in OPN-deficient in vivo glioma models. OPN deficiency also reduces immune-suppressive regulatory T cells in blood and sensitized glioma cells to direct CD8+ T cell cytotoxicity [136]. Tumor-derived OPN stimulates CSF-1 through activation of PI3K/Akt/p65 signaling, leading to the infiltration of macrophages in HCC [137]. Hence, these reports suggested that OPN may induce or maintain M2-type macrophages depending upon pathological conditions and tissue type in cancer.

Single-cell RNA seq (scRNA-seq) analyses reported that OPN is highly expressed in monocyte-derived TAMs compared to resident macrophages in breast cancer [138]. Moreover, TAM subpopulations were divided into two subtypes (Group 1: high expression of CD204, APOE, C1QA, TREM2, CADM1 and OPN; Group 2: strong expression of CD206, S100A9 and FCN1) [139]. Similarly, pan-cancer scRNA-seq data revealed that OPN+ TAMs were predominantly enriched in association with EMT, hypoxia and angiogenesis and enhance tumor metastasis [140]. The single-cell and spatial analyses suggest that FAP+ fibroblasts and OPN+ macrophages cooperate to create a desmoplastic milieu that hinders lymphocytes from penetrating the tumor core, hence decreasing the effectiveness of PD-L1 therapy [8]. At single cell level, OPN+ TAM subsets regulate gene signatures and serve as a novel characteristic marker for M2 TAM. This is associated with the worst prognosis, poor immune cell infiltration and decreased immune checkpoint expression.

**Figure 6:**
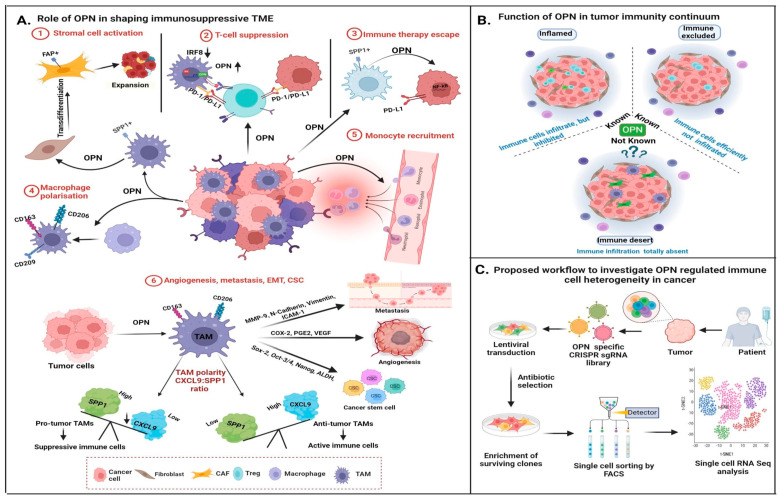
Model depicting the role of OPN in tumor immune microenvironment (TIME): (**A**) Role of OPN in shaping immunosuppressive TME: tumor-derived OPN activates stromal cells by trans-differentiation of fibroblasts to myofibroblasts, resulting in expansion of tumor. OPN-regulated PD-1/PDL1 interaction inhibits T-cell activation. Further, tumor cells downregulate the expression of IRF8, resulting in overexpression of OPN, thus leading to T-cell suppression. OPN, via the NF-κB pathway, upregulates PD-L1 expression, aiding in immune therapy escape. OPN induces the polarization of macrophages and the recruitment of monocytes. It activates TAM, leading to angiogenesis, metastasis and enrichment of CSCs via upregulation of various tumor-promoting factors such as MMP-9, N-cadherin, vimentin, ICAM-1, COX-2, PGE-2, VEGF, Sox-2, Oct-3/4, Nanog and ALDH. The polarity ratio of CXCL9 and OPN (SPP1) determines the anti- and pro-tumorigenic properties of TAMs. (**B**) Involvement of OPN in tumor immunity continuum: OPN is primarily associated with inflamed and immune-excluded tumors, whereas its role in immune-desert tumor remains elusive. (**C**) Schematic representations to identify OPN-regulated immune cell heterogeneity in cancer: OPN-regulated immune modulatory genes may be identified in TIME by CRISPR technology in breast cancer using scRNA-seq based platform.

Lipid-associated macrophages, also known as foamy macrophages, are TAMs that are linked to breast cancer and display an M2-like gene profile, such as CD163 expression, and release different pro-tumor secretory factors [141]. Moreover, OPN has been reported to modulate the expression of IL6 and IL12, suppress the expression of IL27 in dendritic cells, downregulate the expression of IL10 in monocytes, and function as a chemoattractant cytokine that recruits neutrophils and macrophages [6]. Furthermore, a different study found that the NLRP3 TAM, OPN TAM and the IL4I1 TAM niche are strongly linked with tumor nests that include acute inflammation, hypoxia and diffuse tissue necrosis. Bill et al. recently demonstrated the synergistic ratio of CXCL9 and OPN in TAMs [142]. This ratio not only defines TAM (beyond its M1 and M2) polarity but also collectively dictates patient outcomes, and the anti-tumor potential and immunosuppressive behavior of the TME [142] (Figure 6A).

### 6.2. Role of OPN in TAM-Mediated Tumor Progression

OPN-mediated interaction between tumor and macrophages in the TME plays an important role in the promotion of various hallmarks of cancer including proliferation, angiogenesis, metastasis, evading immune destruction and CSC enrichment. OPN induces tumor fibrosis by acting as a promoter of both TAMs and CAFs. CAFs recruit monocytes in tumors and help in polarization into TAMs, whereas TAMs promote activation and proliferation of fibroblasts by secreting TGF-β and PDGFs [143]. OPN acts as a facilitator of these processes in the TME. Tokuda et al. reported that TAM-derived OPN activates hepatic stellate cells into CAFs and promotes malignancy in HCC [144]. Further, crosstalk between tumor cells and TAMs via OPN leads to tumor growth, angiogenesis and metastasis. The reciprocal crosstalk between TAMs and cancer cells via OPN/CD44 axis advances the tumorigenicity through activation of the JNK pathway in colorectal carcinoma [31]. Similarly, Nakajima et al. showed that TAM-derived OPN acts as a key regulator of cancer progression through interacting with CD44v6 in colorectal cancer [145]. Kale et al. demonstrated that tumor-derived OPN promotes macrophage-dependent tube formation ability of HUVEC by inducing COX-2 expression in macrophages via the ERK/p38-dependent signaling pathway in melanoma [64]. Furthermore, ADAM8 enhances the angiogenic potential of macrophages by inducing OPN expression via JAK/STAT3 and NF-κB signaling in glioblastoma [66]. TAMs also function as regulators of stem cell enrichment and maintenance and thus contribute to chemotherapy resistance and tumor relapse. Radharani et al. demonstrated that macrophages activated by treatment with cancer cell condition media positively regulate CSC-mediated tumor progression by IL-6-dependent activation of the JAK/STAT pathway in breast cancer cells [146]. OPN acts as a mediator of crosstalk between TAMs and CSCs and plays a significant role in maintenance of stemness in cancer. It has been also demonstrated that TAMs interact with CD44 in CSCs through OPN to regulate CSC-mediated tumor progression by activating the PI3K/Akt in colorectal carcinoma [101].

### 6.3. Role of OPN in Immune Evasion

Immune evasion is a key hallmark of cancer. Tumors employ various mechanisms to evade immune attack, including restricting antigen recognition, inhibiting the immune system, inducing T cell exhaustion and reprogramming immune cells from tumor suppressor to promoter type. Cancer cells hijack inflammatory mechanisms to convert anti-tumor to tumor-promoting immune cells, which secrete pro-tumor factors that support tumor growth and metastasis. Cancer cells also express immune checkpoint proteins to induce inhibitory signals, leading to suppression of T cell activity [147]. Cancer cells utilize different molecules and signaling pathways to modulate immune response in TIME. OPN has been identified as one such regulatory molecule that can modulate immune response in favor of tumor promotion. It is predominantly expressed in tumors and performs an important role in immune evasion in cancer. OPN plays a regulatory role in T cell activation, conversion of macrophages from M1 to M2 type and expression of immune checkpoint proteins [16].

### 6.4. OPN Regulates T-Cell Activation

OPN is also known as the early T cell-activated gene (*Eta-1*) and regulate the activation of T cell in various malignancies [3]. OPN modulates the adhesion, migration and activation of inflammatory cells along with T cell differentiation to govern the immune response against infection [6]. However, various reports have demonstrated the role of OPN in suppression of T cell responses in cancer. MDSCs, an immature granulocytic or monocytic myeloid cell population, are known to suppress innate and adaptive immune systems by regulating T and NK cell functions. Granulocytic MDSCs are predominantly found in tumors and associated with decreased expression of interferon regulatory factor-8 (IRF-8) in tumors [148]. MDSCs overexpress OPN and suggested that IRF-8 and OPN are negatively correlated. MDSCs and tumor-derived OPN have been shown to abrogate T cell activation and T cell-mediated IFN-γ secretion via interaction with CD44 on T-cells, leading to poor patient survival [7]. Further, tumor- and host-derived OPN render an immunosuppressive effect in the lung metastatic model of breast cancer. Interestingly, monocytic MDCSs were found to be the primary source of host-derived OPN [149]. Tumor-derived OPN is also associated with the recruitment of MDSCs at tumor sites, which might lead to MDSC-mediated suppression of T-cell activity. Allegrezza et al. indicated that trametinib, a MEK inhibitor, induces anti-tumor T-cell activation by inhibiting tumor-derived OPN-mediated MDSC recruitment in breast cancer [150].

Further, researchers demonstrated that silencing OPN in colon cancer cells leads to a significant increase in the efficacy of the tumor-specific cytotoxic T cells, suggesting an immunosuppressive role of OPN [151]. Macrophage-mediated immunosuppressive effects of OPN were determined in the lung adenocarcinoma model, whereas co-culture of macrophages with cancer cells led to diminished activity of CD4+ T-cells that were rescued upon OPN depletion [152]. TIME may be categorized into three different groups: (i) inflamed type, (ii) immune-excluded type, and (iii) immune-desert type [153]. The role of OPN and its function have been explored in inflamed and immune-excluded TIME whereas its function in immune-desert types need to be explored further (Figure 6B).

### 6.5. OPN Regulates Immune Checkpoints

Immune checkpoint molecules are ligand-receptor complexes that exhibit inhibitory or stimulatory effect upon immune responses [154]. Tumor cells regulate immune checkpoints and evade host immune surveillance, leading to tumor progression. PD-L1 is one such immune checkpoint molecule on tumor cells, which binds with its receptor, PD-1, on T cells and induces T cell dysfunction. It has been demonstrated that TAM-derived OPN increases the expression of PD-L1 in NSCLC via NF-κB signaling, resulting in suppression of the anti-tumor immune response [152]. In another report, PD-L1 expression was upregulated in HCC cells by OPN through induction of the CSF1-CSF1R pathway in macrophages [155]. Zhang et al. showed that OPN upregulates PD-L1 in macrophages to facilitate their polarization and immune escape in lung adenocarcinoma [152]. CRISPR-based approaches may be utilized to identify the OPN-driven immune-modulatory genes in breast cancer using the scRNA-seq platform [142] (Figure 6C).

## 7. Osteopontin as a Therapeutic Target

**Figure 7:**
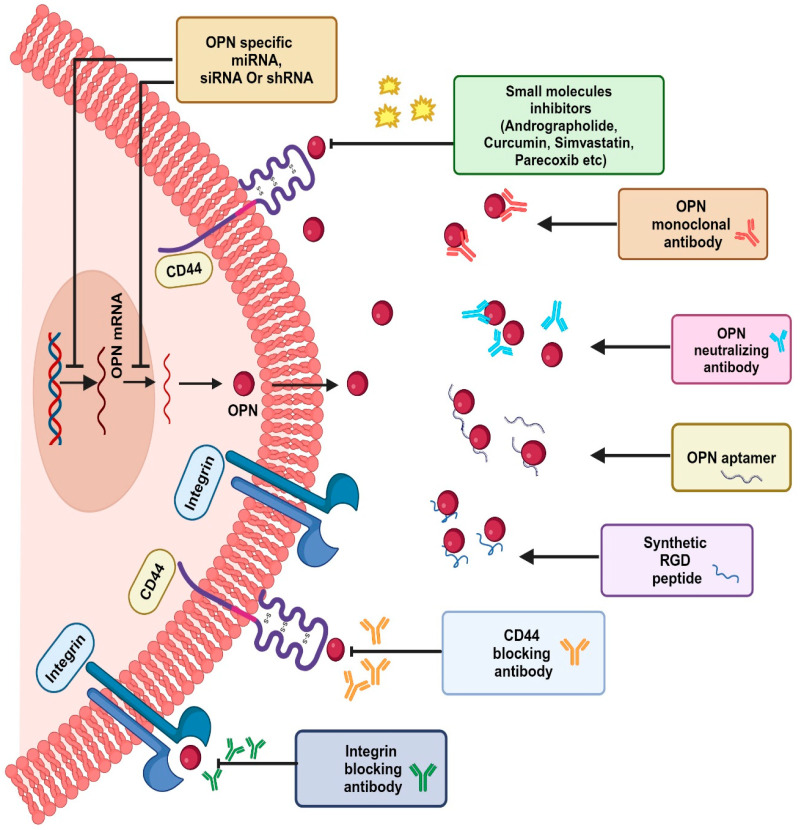
OPN-targeted novel therapeutic strategies. OPN-specific siRNA, miRNA, shRNA, Small molecule inhibitors (Andrographolide, Curcumin, etc.), OPN neutralizing antibodies, OPN aptamers, synthetic RGD peptides, and CD44 and integrin blocking antibodies have been used recently as therapeutic approaches to target the OPN-integrin/CD44 axis, which leads to downregulation of various oncogenic molecules and suppression of tumor progression by disrupting the OPN-regulated signaling pathways in cancers.

Several therapeutic approaches have been reported in targeting OPN, including blocking of the upstream and downstream pathways, inhibition of OPN expression, immune checkpoint blockade and OPN inhibitors (Figure 7).

### 7.1. OPN-Neutralizing Antibody-Mediated Cancer Therapy

OPN-neutralizing antibodies or synthetic peptide binds with OPN or its receptor CD44 or αvβ3 to inhibit the OPN-mediated cellular function [156]. A study in breast cancer cohorts showed a reduction in bone metastasis and osteoclast differentiation after treatment with OPN-neutralizing antibodies along with decreased precursors of osteoclasts [107]. A humanized OPN antibody, hu1A12, recognizes the epitope N^212^APSD^216^ in full-length OPN adjacent to the calcium-binding domain and inhibits cell adhesion and migration in breast cancer [157]. The antibody also exhibited efficacy in reducing the primary tumor growth and spontaneous metastasis using in vivo lung metastatic mouse models [157]. The OPN-neutralizing antibody attenuates Slug-mediated tumor-enhancing ability in CRC patients [158]. The anti-OPN monoclonal antibody AOM1 blocks the αvβ3 binding site as well as the thrombin cleavage site of OPN, thus effectively inhibiting OPN-αvβ3 integrin interaction and reducing cell migration in colon cancer [151]. Similarly, AOM1 was shown to prevent the tumor growth in metastatic lesions of an NSCLC mouse model, while no effect was observed at primary sites [159]. OPN-neutralizing monoclonal antibodies (100D3 and 103D6) reduced tumor growth by attenuating the interaction of OPN with T cells in a colon cancer model [151]. Blocking the interaction of OPN with αvβ3 integrin resulted in decreased expression of MMP-2, ILK and uPA, whereas blocking CD44 interaction resulted in decreased tumor growth in mouse mammary epithelial cancer cells [11]. However, it has been reported that OPN undergoes frequent turnover in healthy human cohorts. The high concentration of OPN in plasma, coupled with its rapid turnover, underscores the demand for enhanced therapeutic antibodies targeting OPN. These antibodies should facilitate high-dose administration within short time intervals and exhibit extended pharmacokinetics compared to conventional antibodies. Thus, antibodies targeting OPN receptors, CD44 or integrin could be administered for OPN-targeted cancer therapy.

### 7.2. Small Molecule Inhibitors as a Potential Therapeutic Agent

Owing to their small volume and easy access to tumor sites, small molecule inhibitors have gained much importance in this current era of cancer therapeutics. One of the well-known small molecules, andrographolide, exhibits its activity via suppressing c-Jun and downregulating the PI3K/Akt signaling pathway, thereby abrogating the expression of OPN and reducing breast tumor growth [160]. Bandopadhyay et al. have reviewed that the expression of OPN is attenuated by HMGR (3-hydroxy-3-methylglutaryl CoA reductase) inhibitor in ovarian cancer, whereas parecoxib, a COX-2 inhibitor suppresses the OPN expression via NR4A2/Wnt pathway thereby reducing the tumor growth in colorectal cancer [11]. It has been reported that luteolin suppresses the expression of OPN in HCC models [161]. Additionally, administration of bisphosphonate resulted in decreased expression of CD44/MMP-9 as well as reduced migration in prostate cancer cells. Bisphosphonate inhibits Rho GTPase activity through disrupting the interaction of OPN with αvβ3 integrin [162].

### 7.3. Epigenetic and miRNA-Based Approaches

Epigenetic alterations can influence the growth and development of healthy cells, leading to neoplastic transformation. Interestingly, the WDR5-H3K4me3 epigenetic axis modulates OPN expression, leading to tumor immune evasion and anti-PD-1 immunotherapy escape in pancreatic cancer [163]. In addition, OPN induces DNA methylation via DNMT1 and renders the CD133+/CD44+ CSC subpopulation more sensitive to 5-azacytidine in HCC [164]. Thus, the OPN-DNMT1 axis promotes aberrant DNA methylation while inhibiting CD133^+^/CD44^+^ sphere formation and migration. These findings suggest that OPN could be an appealing target for HCC resistance through methylome reprogramming [164]. Bromodomain and extra-terminal domain (BET) inhibitors impeded cell proliferation, invasion and migration via decreased OPN expression through transcriptional inactivation of NF-κB [165]. Epigenetically generated splice variants of OPN were observed to confer chemoresistance in colorectal cancer. It has been reported that OPN-c promotes chemoresistance to 5-fluorouracil treatment and the splicing event is regulated by phosphorylation at the S421 site of methyl-CpG binding protein 2 [166].

It is also observed that miR-181c downregulates the expression of OPN, thereby enhancing the chemosensitivity to adriamycin and decreasing chemoresistance in breast cancer cells [167]. OPN siRNA-encapsulated nanoparticles significantly downregulated the OPN mRNA level along with enhanced inhibition of tumor growth in mouse mammary carcinoma models [168]. miR-196a knockout showed decreased expression of OPN and reduced lung metastasis in HCC [169]. Therefore, the identification and combination of epigenetic modulators of OPN with the conventional chemotherapeutic agents offer great potential in preventing tumor growth and recurrence.

### 7.4. OPN Aptamer

Aptamers are highly stable, 12–30 short ssRNA nucleotide sequences that are capable of adopting the 3D structure of the target molecule so as to precisely bind with the protein ligands or small molecules [170]. The well-known OPN aptamer OPN-R3 is tailored to bind with OPN and decrease the cellular migration, invasion and adhesion in MDA-MB-231 cells [171]. In addition, the modified OPN-R3 aptamer has been shown to reduce breast tumor growth using in vivo models [172]. Similarly, OPN-R3 inhibits the binding of OPN to its receptors αvβ3 and CD44, resulting in a decrease of in vitro cell adhesion and invasion [173]. Moreover, it attenuated distant metastasis and tumor progression using in vivo breast cancer xenograft models [173]. Further, the OPN-R3 showed half-life of 7.8 h and localized extracellularly, resulting in breast tumor growth reversal by ablating OPN binding to its receptors [172].

### 7.5. Biomarker

Biomarkers constitute an evolving and dynamic strategy within the realm of cancer research. Numerous discoveries in biomarker identification have significantly contributed to the realms of cancer diagnosis, assessment of cancer progression and monitoring the risk of post-treatment recurrence.

Interestingly, it is found that expression of OPN is a predictable biomarker not only for breast cancer but also for NSCLC, HCC, gastric, prostate, colorectal and other cancers [102,165,174,175,176,177,178,179]. Levels of OPN expression can be effectively used to evaluate the histological grade of tumors, clinical stage, response to treatment, risk of recurrence after surgery, overall survival and disease-free survival [10]. OPN’s efficacy as a prognostic and diagnostic biomarker in various cancer types is summarized in Table 2.

## 8. Conclusions and Future Perspective

OPN emerges as a pivotal regulator in the intricate interplay between cancer cells and the TME, exerting influence across various facets of cancer progression. The abundance of OPN within the TME is responsible for modulating the fate of tumor and stromal cells but its clinical relevance remains a major limiting factor. Our earlier reports have dissected several OPN-modulated fundamental signaling pathways in various cancers [3,11,63,65]. Furthermore, its multifaceted role encompasses angiogenesis, CSCs, bone metastasis, cancer cell metabolism and modulation of the TME, involving interactions with CAFs, adipocytes, osteoclasts and immune cells. In addition, OPN intricately regulates cancer cell metabolism, particularly in glucose metabolism, influencing glucose transporters and contributing to the Warburg effect [111]. Moreover, OPN plays a central role by affecting the bone microenvironment, promoting bone metastasis and contributing to the formation of premetastatic niches [98]. Thus, OPN acts as a potential therapeutic target for mitigating osteoclast development and controlling skeletal metastases.

In the TME, the influence of OPN spans interactions with CAFs, adipocytes and osteoclasts. OPN-reprogrammed CAFs contribute to proinflammatory states, angiogenesis and EMT, thereby emphasizing its role in cancer progression [63]. OPN significantly impacts immunomodulation within the TME, influencing macrophage polarization towards a tumor-promoting phenotype (M2 type) and contributing to an immunosuppressive microenvironment. Moreover, OPN is implicated in immune evasion mechanisms, including the inhibition of T-cell activation and the regulation of immune checkpoint molecules such as PD-L1. CAF and TAM interactions are majorly regulated by OPN and its associated signaling events, causing upregulation of various tumor events. For example, scRNA-seq revealed that OPN mediates the interaction between OPN-PTGER4 and OPN-CD44, stimulating the crosstalk between HCC cells and macrophages [180]. Similarly, single-cell transcriptome sequencing identified three TAM subgroups: C1Q+, FCN1+ and OPN+ TAMs where OPN+ TAMs modulate the TIME via interaction with CAFs [181]. Zhang et al. reported that OPN+ TAMs are tightly associated with CAFs and endothelial cells in modulating the TME [182]. However, the complex molecular mechanism of OPN-mediated TAM subset enrichment, regulation of metabolic switch and intricate crosstalk between CAF-TAM is poorly elucidated. Although multiple studies have demonstrated the function of CAFs in regulation of TAM, comprehending the influence of TAM in controlling CAF phenotypes warrants further investigation.

Single-cell and spatial transcriptomics, immunofluorescent labeling and other methods have advanced our understanding of OPN in inflamed and the immune-excluded TME. However, its role in the immune desert needs further exploration [153] (Figure 6B). Detailed mechanistic studies on OPN-regulated networks and core regulatory transcription factors governing OPN expression in tumor and immune cells are warranted.

Considering the central role of OPN in cancer progression, targeted therapies in blocking OPN expression or its downstream signaling pathways hold promise. However, current interventions lack efficacy in both preclinical and clinical trials, necessitating further development of small molecules or antibodies to neutralize the effect of OPN. Stratifying cancer patients based on expression levels of OPN and associated signaling pathways could tailor treatment strategies and identify the patient subgroups more responsive to OPN-targeted therapies. Understanding the immunomodulatory role of OPN suggests exploring combination therapies involving OPN inhibition and immunotherapeutic agents, potentially enhancing the effectiveness of immune checkpoint inhibitors and promoting antitumor immune responses.

In summary, the diverse functions of OPN in cancer underscore its significance as a potential therapeutic target and diagnostic marker. Thus, the continued research on the role of OPN and its associated signaling networks will unravel new insights in developing innovative therapeutic strategies for precision medicine, ultimately improving patient outcomes in various cancers.

## Figures and Tables

**Table 1:** OPN-mediated signaling pathway in various cancers.

Cancer Type	Pathway	Function	References
Colorectal	PI3K-Akt-GSK3/ß-catenin pathway	Cell proliferation, migration and invasion	[32]
Oral	PI3K/AKT/mTOR pathway	Cell proliferation, invasion, metastasis and angiogenesis	[34]
Breast	JAK2/STAT3	Apoptosis and migration	[53]
Prostate	Akt/mTOR and MNK/eIF4E	Immune evasion and metastasis	[54]
Bladder	JAK1/STAT1 pathway	Apoptosis, proliferation	[55]
Gastric	PI3-K-Akt and NF-κB pathways	ECM degradation, migration and cell proliferation	[56]
Gastric	MAPK, PI3K and NF-κB	ECM degradation, metastasis and apoptosis	[57]
Hepatocellular carcinoma	JAK2/STAT3/NOX1 signaling	Proliferation, ECM degradation and migration	[58]
Ovarian	PI3K/AKT signalling	Chemoresistance	[59]
Head and neck	Integrin αvβ3-NF-kappa B pathway	Cell proliferation, migration, invasion and stemness	[60]
Non-small cell lung	OPN/integrin αvβ3/FAK signallingin NSCLC	Promote cell proliferation	[61]
Non-small cell lung	NF-κB pathway in NSCLC	Metastasis, proliferation and immunosuppression	[62]
Breast	Akt/Erk-1 pathway	EMT, metastasis and angiogenesis	[63]
Melanoma	ERK-1/Akt/AP-1 pathway	Macrophage polarization, metastasis, angiogenesis,	[64]
Breast	Brk/NF-kB/ATF-4	Angiogenesis	[65]
Glioblastoma	JAK/STAT3 pathway	Angiogenesis	[66]

**Table 2:** OPN as early prognostic and diagnostic biomarker in various cancers.

Cancer Type	Biomarker	Clinical Significance	Prediction Type	References
Gastric	OPN	High OPN expression associated with lymph node metastasis, TNM stage, depth of invasion, tumor size and distant metastasis	Prognostic	[102]
Melanoma	OPN	OPN overexpression associated with poor prognosis	Prognostic and diagnostic	[165]
Breast	OPN + COX-2	Overexpression of OPN and COX-2 indicates poor prognosis	Prognostic	[174]
Breast	OPN-c + E-cadherin + β-catenin	OPN-C expression correlated with TNM staging and histological grading	Diagnostic (staging and grading)	[174]
Non-small cell lung	OPN + VEGF	OPN and VEGF positive shows worse prognosis	Prognostic	[174]
Non-small cell lung	OPN, CD44v6 and MMP-2	Overexpression of OPN, CD44v6 and MMP-2 associated with staging and histology	Diagnostic	[174]
Gastric	OPN, E-cadherin, β-catenin	Overexpression of OPN, E-cadherin and β-catenin serve as prognostic factors	Prognostic	[174]
Renal cell carcinoma	OPN (with PAZ treatment)	High concentrations of six CAFs signature IL-6, IL-8, HGF, OPN, VEGF-A and TIMP-1	Prognostic	[175]
Malignant pleural mesothelioma (MPM)	OPN	OPN as diagnostic marker for MPM	Diagnostic	[176]
Colorectal	OPN	Associated with high tumor grades and metastasis	Prognostic	[177]
Hepatocellular carcinoma	OPN	Serum/plasma-based OPN have significant predictive ability and diagnostic value	Prognostic and diagnostic	[178]
Cervical	High OPN, low E-cadherin or both	Resistant to radiotherapy, negative prognostic factor for patient survival	Prognostic	[179]
